# Innovative uses of communication technology for HIV programming for men who have sex with men and transgender persons^1^

**DOI:** 10.7448/IAS.17.1.19041

**Published:** 2014-10-01

**Authors:** Susannah M Allison, Darrin Adams, Kent C Klindera, Tonia Poteat, R Cameron Wolf

**Affiliations:** 1 Division of AIDS Research, National Institute of Mental Health, National Institutes of Health, Bethesda, MD, USA; 2 Health Policy Project, Futures Group, Washington, DC, USA; 3 GMT Initiative, amfAR–The Foundation for AIDS Research, New York, NY, USA; 4 Johns Hopkins Bloomberg School of Public Health, Baltimore, MD, USA; 5 U.S. Agency for International Development, Washington, DC, USA

**Keywords:** MSM, transgender, communication technology, HIV prevention, HIV care, mHealth

## Abstract

Globally, overall rates of HIV are on the decline; however, rates among gay men and other men who have sex with men (MSM) and transgender persons are increasing. Meanwhile, there has been exponential growth in access to communication technology over the last decade. More innovative prevention and care technology-based programmes are needed to help address the growing numbers of MSM and transgender persons living with HIV and those at risk for infection. To address this need, a meeting was hosted by the U.S. Agency for International Development (USAID) through the President's Emergency Plan for AIDS Relief (PEPFAR) and co-sponsored by amfAR, The Foundation for AIDS Research and the National Institute of Mental Health (NIMH). The meeting brought together researchers, community implementers, advocates and federal partners to discuss the current landscape of technology-based interventions for MSM and transgender persons and to discuss key considerations. Presentations and discussions focused on the research gaps, facilitators and barriers to programme implementation and public–private partnerships. This article summarizes the meeting proceedings and outlines key considerations for future work in this area.

## Introduction

There is an increasing awareness of the high prevalence rates of HIV among men who have sex with men (MSM) and transgender persons globally [1,2]. In most countries throughout the world, MSM and transgender women carry a higher burden of HIV compared to others in the general population [1,3]. While global incidence data are scant among MSM and almost non-existent among transgender populations, this body of evidence is growing.

### HIV among MSM

Data are emerging on the HIV epidemics among MSM in low- and middle-income countries. In Kenya, Malawi and Thailand, HIV incidence over a one-year period among MSM has been found to be 8.6, 7.1, and 5.9%, respectively [4–6]. Incidence rates may be higher among sub-samples of MSM who report having sex exclusively with men. For example in a further analysis of data from Kenya, a high incidence of 35.2 per 100 person-years was shown among these men who only have sex with men, many of whom reported receiving money for sex [4]. The high rates could also be attributed to the fact that the researchers were tapping into an inter-connected network of adults with ongoing high-risk sexual activity.

In some high-income countries, overall new infections have been on the decline, yet among MSM they have been rising. This is particularly true among young black MSM in the United States [2,7–9]. Millett and colleagues [10] found black MSM more likely to be HIV positive and less likely to initiate antiretroviral therapy when compared to other MSM, despite being more likely to report HIV prevention behaviour. Additionally, the UK [11], Western Europe [2], Australia [12] and sub-Saharan Africa [13] have all seen increases in HIV incidence among MSM in the past few years.

While our understanding of HIV epidemiology among MSM in low- and middle-income countries is improving, critical gaps remain in our ability to reach MSM with effective interventions. In an online survey of over 3700 MSM in over 140 countries, Ayala and colleagues [14] discovered low access to HIV testing (35%), treatment (43%), and HIV prevention commodities such as condoms (35%) and condom-compatible lubricants (22%). In a comprehensive review of studies of HIV prevention interventions worldwide, Sullivan [15] also found that prevention efforts only reach a small proportion of the MSM population. Key messages from this work are that coordinated combination prevention interventions that incorporate behavioural, biomedical, and structural components are needed to reduce HIV incidence among MSM and that scalability should be considered when new combination interventions and approaches are developed.

### HIV among transgender persons

Compared to the small yet growing amount of data on the HIV epidemic among MSM worldwide, much less is known about the HIV epidemic among transgender persons. Most of the existing data focuses on transgender women and little has been published on HIV among transgender men, who may also have sex with other men. Studies on transgender women indicate that they bear a high burden of HIV. In a systematic review of research studies in the United States, transgender women were found to have an average HIV prevalence of 27.7% in the four studies that used laboratory-confirmation of HIV status [16]. A lower average HIV prevalence (11.8%) was found in the 18 studies that relied on self-report, suggesting that many transgender women may be unaware of their HIV status. In a more recent meta-analysis of research conducted worldwide [3], transgender women were found to have an overall HIV prevalence of 19.1%, 48.8 times the odds of laboratory-confirmed HIV infection when compared to adults in the general population. This disproportionate burden was found in both middle- and high-income countries. At that time, no published data on HIV among transgender women was found from sub-Saharan Africa, Eastern Europe, or Central Asia [3].

Despite the burden of HIV among transgender women, few evidence-based interventions have been evaluated specifically among this population [17,18]. HIV prevention and care clinical trials sometimes enrol transgender women along with MSM [19,20]. However, the number of transgender participants is often too small for meaningful disaggregated analysis. Transgender populations are diverse both within and between cultures and contexts; therefore, understanding both the differences between MSM and transgender populations as well as the heterogeneity within each group is critical to the development of appropriate interventions and their effective implementation.

### Growth and access to communication technology

Use of newer forms of communication technology (e.g. Internet use whether through a computer or mobile phone, SMS, smartphone apps) has grown exponentially over the last few years. Since 2005, the number of people using the Internet globally has more than doubled, going from 16 to 39% [21]. There are currently almost as many mobile–cellular subscriptions as people in the world [21]. While use of new communication technologies has increased, there are still significant differences between and within countries regarding access. For this reason, it is important to understand what forms of communication technology populations of interest are using, how they access and use the technology, and what barriers and facilitators exist to their use (i.e. cost, connectivity, phone capabilities, etc.).

Some data suggest that MSM use new forms of technology at even higher rates than the general population [22–25]. Additionally, significant numbers of MSM use the Internet and mobile apps to find romantic and sexual partners [26]. Unfortunately little data exists on technology use among transgender populations. We, therefore, need to have a greater understanding of the way that MSM and transgender persons use new forms of technology and more work needs to be done to harness the power of these technologies for HIV prevention and care.

## Discussion

Public health officials are deeply concerned about the gap that exists globally between the need for HIV prevention and care services for MSM and transgender persons and what is available. In response to this critical situation, a group of more than 40 prominent HIV activists, scientists, entrepreneurs, and public health leaders from around the world met to discuss how Internet, social media, and other forms of communication technology can improve the impact of HIV programmes for MSM and transgender persons. Meeting participants included representatives from Africa, Latin America, the Caribbean, Asia, Europe, Australia and the US. The goal of the meeting, hosted by U.S. Agency for International Development (USAID) through President's Emergency Plan for AIDS Relief (PEPFAR) funds and co-sponsored by amfAR, The Foundation for AIDS Research, and National Institute of Mental Health (NIMH), was to provide a forum for key stakeholders in HIV research, programming, implementation and evaluation to take stock of important developments in the field and develop key considerations to enhance the use of communication technologies in the delivery of HIV prevention and care for MSM and transgender persons with a specific focus on low- and middle-income countries.

### Innovative programmes for MSM and transgender persons

The meeting highlighted a number of innovative programmes that are utilizing the power and reach of communication technology to impact the HIV epidemics among MSM and transgender persons around the world. While the meeting could not include representatives from every successful programme doing work in this area, the programmes highlighted at the meeting (Table 1) reflect a range of innovative programming from different regions around the world. The selected programmes were identified through a scoping exercise, which sought to compile innovative and diverse practices and experiences from community-based organizations, private sector partners, academic institutions and government programmes. Please also see the meeting website for the full meeting report and presentations from the speakers [28]. Many of the programmes have not been rigorously evaluated to date; however, qualitative analyses and process evaluations have shown a number of programmes to be promising practices in addressing components of the HIV prevention, care and treatment continuum for MSM and transgender persons.

**Table 1:** Examples of programmes for MSM and transgender persons utilizing innovative communication technology

Programme/organization	Country	Presenter	Description of programme	Innovative communication technology	Links for more information
Adam's Love	Thailand	Tarandeep Anand	Partnership with the Thai Red Cross Society; innovative use of social marketing strategies to promote sexual health among MSM including HIV testing; successfully used male celebrities as models	Website Online chat rooms Social networking sites	www.adamslove.org
AIDS Council of New South Wales	Australia	Yves Calmette	“Test More+Treat Early+Stay Safe=Ending HIV” media campaign; combination of traditional print marketing with online strategies	Social networking sites Website	endinghiv.org.au
Jamaica Youth Advocacy Network	Jamaica	Javion Nelson	I live out Loud! web-based programme promotes youth activism on public policy to motivate LGBT youth	Website	www.iliveoutloud.org
Be Change Foundation	Philippines	Laurindo Garcia	Website that provides a platform for activists and programme implementers to share ideas and strategies	Website	www.hivadvocates.net
Text Me! Flash Me! Call Me! By FHI360	Ghana	Jacob Larbi	Website, telephone helpline, online chat room, and SMS messages and reminders to increase HIV testing and treatment adherence	Website Online chat rooms SMS	http://www.comminit.com/content/text-me-flash-me-helpline
Alternatives/Cameroun	Cameroon	Yves Yomb	Online outreach on social networking sites to offer HIV prevention messages and encourage HIV testing	Social networking sites	http://www.amfar.org/content.aspx?id=9273
Thai Ladyboyz	Thailand	Nada Chaiyajit	Uses social networking sites to promote health-seeking behaviour and provide mental health support for transgender persons	Social networking sites	https://www.facebook.com/TLBzSexperts [27]
Jamaican Forum for Lesbians, All-Sexuals and Gays	Jamaica	Javion Nelson	YouTube campaign to promote tolerance and respect for the LGBT community	YouTube	www.youtube.com/user/EqualityJA
Silueta X	Ecuador	Diane Rodriquez	Use social networking sites, e-blasts and their website to address transgender rights and health	Social networking sites Email Website	Siluetax.wordpress.com

Programmes highlighted at the meeting are reaching out to MSM and transgender persons both online and offline. Successful social marketing campaigns in Thailand and Sydney, Australia (Adam's Love and AIDS Council of New South Wales (ACON)) have effectively combined traditional print media with online strategies. Traditional print media drive MSM to online blogs, videos and social networking sites where they become more engaged in the campaign, increase their knowledge about sexual health and can link to sexual health services. Organizations have also successfully launched online media campaigns to promote activism among MSM and LGBT community members with the idea that empowered individuals will engage in less risky behaviour. The Jamaica Youth Advocacy Network and B-Change Foundation both provide platforms for activists to share ideas and strategies as well as advocate for policy changes.

Instead of building new online infrastructures, programmes such as Alternatives-Cameroun, Silueta X, and the ThaiLadyboyz provide peer outreach on already established social networking sites that are popular with their populations of focus. The majority of programmes represented at the meeting use Facebook and YouTube to further promote their mission. For example, the Jamaican Forum for Lesbians, All-Sexuals and Gays (J-FLAG) launched a successful YouTube campaign to promote tolerance and respect for the LGBT community.

Building on the increasing access to mobile phones around the world, programmes are beginning to incorporate telephone helplines and SMS messaging into their list of services. SMS messages are used to promote HIV testing and HIV treatment adherence. One example is the Text Me! Flash Me! Call Me! programme in Ghana, designed to increase access to friendly and confidential HIV/AIDS information, referrals and counselling services.

Lastly, a number of programmes have introduced non-financial incentives as a way to increase engagement in sexual health services. Adam's Love in Thailand successfully included online videos of male celebrities and gay personalities promoting testing into their media campaign (see Table 1 for more information about the specific programmes highlighted at the meeting).

In addition to representation from programme implementers from around the world, the meeting also included individuals from the private sector. In some instances, private sector partners have sent out mass messages to their users. For example, smart-phone-based social networking companies offer health alert “pop-ups,” informing users of specific health concerns in their communities, and ways for users to take action and seek health services, such as testing for HIV. At the meeting, the owner and founder of the social chat and dating site *MISTER* described his company's past partnerships with public health agencies, such as the creation and distribution of alerts regarding a recent meningitis outbreak in New York City.

### Innovative research for MSM and transgender persons

A number of HIV prevention and care technology-based interventions have been developed for MSM, including those using the Internet [29,30], SMS [31–34] and mobile phone applications [35]. Given the growing amount of research in this area, a list of key principles and lessons learned in the use of emerging technologies for HIV prevention for MSM was recently developed [36]. Recommendations included the development of standards for reporting results of online surveys, strategies to avoid the over-surveying of MSM online, and improved streamlined funding mechanisms to keep up with technology. The majority of interventions have been developed and tested in higher income countries, such as the US and Australia. While the literature is growing on technology-based interventions in low- and middle-income countries [37–39], few studies in these settings focus on the unique HIV prevention and care needs of MSM and transgender persons [40]. While technology-based HIV prevention and care programmes exist for transgender populations and are currently being evaluated, at this time, the authors were unable to find any published results of interventions in this area. This represents a significant gap in our understanding of how best to address the unique needs of this population around the globe.

Several NIH-funded investigators presented at the meeting on their research in this area (see Table 2 for more information about these four researchers). Data on novel web-based interventions to reduce HIV risk behaviour, increase HIV testing and increase sexual communication among MSM were described [41,43]. An online, interactive HIV prevention programme for young MSM is engaging for youth and has an impact on unprotected sex [41]. Strategies are also being developed to reduce stigma, social isolation and sexual risk among young black MSM (YBMSM) [35]. One research group is currently developing a virtual community for YBMSM in North Carolina to address these issues [42]. Lastly, research was presented on work that has been carried out to identify low-cost, online strategies to reduce HIV transmission that would be supported by three stakeholder groups: HIV public health experts, social networking website owners and MSM users of social networking sites [44]. A majority of the three stakeholder groups expressed high levels of support for eight different strategies, including allowing users to filter partners by their profile information, including an online STD testing directory and automatic reminders at intervals of users’ choice to get tested.

**Table 2:** Examples of technology-based HIV prevention research intervention studies for MSM

Principle investigator	University	Description of intervention	Reference^a^
Brian Mustanski	Northwestern University	Keep it Up!, an online STI/HIV prevention programme for young, recently tested, negative MSM. The intervention addresses peer and social norms, personal risk, the danger of making assumptions about HIV status and assertive communication through the use of videos, games, and animation.	[40]
Lisa Hightow-Weidman	University of North Carolina, Chapel Hill	HealthMpowerment, an online virtual community and behavioural intervention for young black MSM in North Carolina. The virtual community is intended to help meet the needs of YBMSM to potentially reduce social isolation, establish positive behavioural norms, and improve health outcomes.	[41]
B.R. Simon Rosser	University of Minnesota School of Public Health	SexPulse, an Internet-based sexual health promotion intervention for MSM. Different modules help users build a comprehensive portrait of their sexual health. Modules address: body image, factors that contribute to risk-taking or risk-avoidant behaviours, clear communication online, and the impact of intimacy or other emotional issues on decision making.	[42]
Dan Wohlfeiler	University of California, San Francisco	Research to build consensus between owners of dating websites, users of these websites and HIV/STD directors; identified eight strategies with high buy in by all three groups of stakeholders.	[43]

a For more information about these studies (how participants were recruited, what was included in the various interventions, etc.), please refer to the references listed here.

## Key considerations from the meeting

Interspersed throughout the two day meeting was time for attendees to discuss the presentations, talk about lessons learned from their own work and think about next steps. These discussions along with the presentations highlighted important key considerations that need to be incorporated into future work utilizing communication technology in order to address the HIV prevention and care needs of MSM and transgender persons (Table 3). Many of these key considerations apply to researchers, programme implementers and donors.

**Table 3:** Key considerations for future research, programming and funding utilizing communication technology to address the HIV prevention and care needs of MSM and transgender persons

Community mobilization and engagement are vital Recognize the variety of communities with which MSM and transgender persons are involved and collaborate with both online and offline community members to ensure adequate community representation. Given the inherent risks to privacy associated with using ICT, community members need to be involved in helping to ensure that their safety and privacy is protected. Separate attention needs to be given to transgender persons and MSM Separate attention needs to be given to the ICT and HIV prevention and care needs of transgender persons and MSM. Need to foster more public–private collaborations Work with the private sector to build on their existing infrastructure and reach. Acknowledge the strengths and weaknesses of both partners in a PPP. Use creative strategies to involve the private sector in HIV prevention and care programmes and research. Address structural issues of homophobia and transphobia Despite the benefits to ICT approaches, homophobia and transphobia can still serve as barriers to these services and need to be acknowledged and steps proactively taken to address them. Combine the virtual with the physical Programmes should take advantage of the strengths of services that can be delivered virtually as well as those that can be delivered in person. Dynamic, bidirectional links should be established between the virtual and physical to reinforce behaviour change. Improve Monitoring and Evaluation for technology-based services A working group should be formed to establish an appropriate set of indicators for technology-based services. Creative solutions are needed to establish the links between technology-based services and overall goals of a programme. More transgender targeted technology-based approaches More research is needed on how transgender populations use technology. Develop more transgender-specific HIV prevention and care interventions. More technology-based programmes for MSM and transgender persons living with HIV While technology-based programmes for MSM and transgender persons living with HIV exist, more attention needs to be given to their scale up and evaluation. More attention needs to be given to the heterogeneity within these populations as well as the potential for utilizing virtual social networks to enhance care. Need for technology-based interventions for organizations and health/social service providers More programmes should focus on clinics, organizations and health care/social service providers. Systems level approaches can help to reduce structural barriers to effective care. Greater speed and flexibility in funding and the ethical review of communication technology proposals Innovative mechanisms are needed to expedite IRB review and grant approval processes to allow for innovative technology-based approaches to move forward in a timely manner. More flexible and dynamic partnerships are needed within PEPFAR to allow for the implementation of promising ICT-based services.

## Community mobilization and engagement are vital

Mobilization of transgender and MSM communities and engagement of community members are crucial components in research and programming. Donors, programme implementers and researchers should recognize multiple forms of community. For example, in online social networks, MSM and transgender populations could be thought of as virtual communities who predominantly interact with each other online, only occasionally interacting in person. Additionally, one person or organization cannot represent an entire community, especially when considering online communities. Therefore, efforts should be made to know the various online and offline opinion leaders. Community members are experts in the latest forms of technology being used in their communities to connect with their peers and sexual partners.

Engaging with MSM and transgender persons in online spaces requires a higher sensitivity and awareness of safety, security, and privacy. Potential breaches and consequences could result in online and offline harassment, leaks of personal and private information, exposure of hidden behaviours and identities, violence, and even death. Given the inherent threats that exist when using communication technology, protocols should be developed in collaboration with community members to ensure the protection of both online and offline identities. For example, community members may be more apt to engage on sensitive issues if there is a measure of anonymity available to them (i.e. a HIV-positive MSM and transgender persons chat room support group).

## Disaggregating transgender and MSM

From clinical trials to representation at meetings, transgender communities and individuals are often combined with MSM when it comes to HIV planning, programming, and research. But their needs and experiences, though overlapping in some ways, are quite varied and different in most ways. Programme implementers, researchers, and donors need to address the separateness of these two populations. Donors should design funding initiatives that address the specific health concerns of transgender persons and MSM separately, incorporating community input. Programme implementers and researchers should develop segmented, community-informed programmes and research protocols that assess communication technology use, cost, and feasibility separately for MSM and transgender persons.

## Need to foster more public–private collaborations

Public and private collaborations are needed in order for communication technologies to have a greater impact on the HIV epidemic. Programme implementers and researchers should work more closely with website owners that already have content and social and sexual networking structures in place for MSM and transgender persons. By integrating into structures that already exist and have a wide, dedicated audience, HIV programmes can have a considerably larger, more sustainable reach.

Donors, programme implementers, and researchers have a mixed history of collaborating with website owners and private sector partners, and this history should be noted along with an acknowledgement of the strengths and weaknesses inherent in both sectors. The public sector has its own strengths (e.g. sustained funding and high HIV technical knowledge) and limitations (e.g. bureaucracy and inflexible funding mechanisms). The private sector, on the other hand, can act quickly and knows the demographics and behaviours of its users very well, but they may have to adhere to external regulations (e.g. app store restrictions on sex and sexuality). In addition, the private sector operates from a consumer-driven model, and HIV services and programmes may not always fit into that model. By acknowledging and anticipating these factors, public and private sector partners can mitigate and address issues before and as they arise.

Donors should encourage private sector participation in the development and implementation of innovative HIV programmes and services by providing flexible funding mechanisms and including other incentives like sharing branding and collaborating on documentation. Programme implementers and researchers should incorporate the expertise of the private sector to develop HIV programmes and services. More attention should be made to create incentives that appeal to those in the private sector to participate and engage on HIV prevention and care research and programme advisory committees.

## Address structural issues of homophobia and transphobia

Homophobia and transphobia should be recognized as core factors in denying access to HIV services for MSM and transgender persons. Communication technology approaches offer benefits in addressing these barriers (e.g. ability to engage in online services anonymously, ability for users to connect with gay- and trans-friendly services virtually); however, homophobia and transphobia are still present and play a role in the delivery of communication technology HIV prevention and care services. These issues need to be acknowledged, addressed through cultural competency trainings and inclusion of community members in the planning process, and measured in the implementation of interventions. As mentioned above, engaging MSM and transgender community members is an efficient means to not only address these structural barriers, but also reduce mistrust among community members of researchers’ intentions.

Additionally, implementers and researchers should recognize that community members may not use their real names or may have multiple email and Facebook accounts, reflecting the multiple identities they may have. Funds should be allocated to provide security training to individuals and organizations, as well as funds to develop and implement contingency plans if a breach in security occurs.

## Combine the virtual with the physical

Organizations should be cautious about letting the pendulum swing too far towards providing all of their services online or via mobile phones. There are many benefits to providing services using newer forms of technology; however, programmes that take advantage of the strengths of both virtual and physical services are likely to have a greater impact. Like other effective interventions, communication technology programmes should go beyond providing unidirectional behaviour change communication. Communication technology provides an opportunity for dynamic, bidirectional links between the virtual and the physical as well as providers and clients. For example, online apps may refer users to HIV testing sites; when they get tested, they may then receive an online badge or other virtual acknowledgement.

## Improve monitoring and evaluation for technology-based services

Currently, certain communication technology services provided by organizations are difficult to monitor and may not be recognized by donor reporting mechanisms. For example, visits to a website or number of HIV-related chats in an online chat room are important outputs which can demonstrate the reach of technology-based interventions for key populations. More work needs to be done to incorporate these into donor-funded programme monitoring and evaluation. Attention by PEPFAR and NIH should be given to develop an appropriate set of indicators for technology-based services, setting standards for what counts as “being reached” by donors.

Additionally, more attention needs to be given to evaluating the relationship between the uptake of technology-based services (e.g. number of SMS messages sent) and overall goals of a programme (e.g. increase in HIV testing rates). This is particularly challenging given the inherent anonymity of some online services; creative solutions are needed to evaluate these linkages.

## More transgender targeted technology-based approaches

Given the unique HIV prevention and care needs of transgender populations and the huge health disparities that exist for these populations, more research and programming targeting transgender persons is needed. In particular, more needs to be known about how transgender populations use technology (e.g. do transgender women access HIV prevention information on the web and if so do they access information on sites specifically for gay/bisexual men or heterosexual women or more general HIV prevention sites?). Additionally, innovative transgender-specific HIV prevention and care interventions need to be developed and tested for this population.

## More technology-based programmes for MSM and transgender persons living with HIV

Communication technology is being used to offer vital services for MSM and transgender persons living with HIV—addressing various components within the continuum of prevention, care and treatment. For example, utilizing technologies that remind and encourage clients to take medication, attend health and other social service appointments, as well as tools to inform health providers of problems with adherence, missed appointments, etc. Other technologies such as apps may be useful for mapping, tracking and advertising MSM and transgender-friendly health services. More work should be focused on evaluating and scaling up these interventions. Social networking approaches should also be scaled up as efficient means of working with MSM and transgender persons living with HIV by creating virtual networks that help to reduce feelings of isolation, encourage health-seeking behaviour, and engage individuals in advocacy efforts.

## Need for technology-based interventions for organizations and health/social service providers

With an increasing focus on the use of Health Management Information Systems (HMIS), in particular the use of new technologies to improve patient care, more work needs to focus on the development and testing of communication technology systems and strategies to support quality care for MSM and transgender persons. Researchers and programme implementers are encouraged to move beyond targeting clients and also develop and implement interventions for organizations and health care providers as well as other social service providers. These interventions can help to address some of the structural and systemic barriers to care for MSM and transgender persons. Examples include the provision of web-based sensitivity trainings and electronic record-based interventions to ensure that providers are addressing the health needs of MSM and transgender patients/clients. When intervening at the clinic or organizational level, added attention needs to be paid to ensure the privacy of data within electronic health systems, especially in countries with anti-LGBT laws.

## Greater speed and flexibility in funding and the ethical review of communication technology proposals

Donors should provide flexible mechanisms that allow research and programmes to respond to the fast-paced, dynamic nature of communication technology. Currently, it takes a minimum of nine months from the submission of an NIH application until the grant award is made [45]. It may also take several months for a research proposal to undergo ethical review by institutional review boards (IRBs). By this time, an originally timely communication technology proposal may no longer be relevant. Innovative structures are needed to expedite IRB review and grant approval processes to allow for researchers to capitalize on current communication technology trends. Researchers and programmers should share lessons learned in working with donors and IRBs. Changing these entrenched structures is a significant undertaking; therefore, action is needed now.

Similarly, PEPFAR funding typically has long planning and funding cycles to develop operational plans, which are developed and reviewed long before funding arrives. More flexible and dynamic partnerships are needed to allow timely and responsive innovation in this area, through development or more rapid cycles of pretesting, piloting and evaluating new content and approaches.

## Conclusions

This meeting was a collaborative effort between a number of partners (USAID, PEPFAR, amfAR, The Foundation for AIDS Research, and NIMH). The discussion highlighted the continued need for communication and collaborations between these different agencies and foundations, the programmes and researchers that are carrying out this important work and community members in order to address the HIV epidemic among MSM and transgender persons. We encourage everyone to take into account the key considerations raised during this meeting and enhance our efforts to prevent and care for HIV among MSM and transgender persons around the world, working towards an AIDS-free future. The time is now.
